# Mechanistic study of PD-L1 regulation of metastatic proliferation in non-small cell lung cancer through modulation of IRE1α/XBP-1 signaling pathway in tumor-associated macrophages

**DOI:** 10.18632/aging.206082

**Published:** 2024-08-24

**Authors:** Yi Wang, Lei Yang, Zezheng Liang, Ming Liu

**Affiliations:** 1 Department of Radiation Oncology, Fourth Hospital of Hebei Medical University, Shijiazhuang, China

**Keywords:** PD-L1, macrophage, IRE1α/XBP-1 signaling pathway, non-small cell lung cancer, nivolumab, P-STAT3

## Abstract

Objective: To explore the related research of PD-L1 in IRE1α/XBP-1 signaling pathway on non-small cell lung cancer.

Methods: The tumor model of mice was established and divided into four groups; after successful modeling, the tumor tissue of mice was removed for subsequent experiments; the bought THP-1 cells were grouped into four different groups, a control group, nivolumab intervention group, IRE1α inhibition group, and nivolumab intervention + IRE1α inhibition group; after co-culture of the four groups of THP-1 cells with A549, THP-1 cell protein levels in the four groups were analyzed using Western blot; A549 cell migration, invasion and proliferation were assessed using the scratch assay, Transwell method, monoclonal experiment and CCK-8 method.

Results: *In vivo* studies indicated that the stimulation of nivolumab could strongly check the progress of NSCLC (non-small cell lung); two groups treated with 4 μ8c showed obvious effects on check point of NSCLC; *In vitro* experiments including Western-blot experiment, Scratch experiment, Transwell method, Monoclonal experiment and CCK-8 experiment suggest that nivolumab could inhibit migration, invasion and proliferation of NSCLC tumor cells and it.

Conclusion: PD-L1 is capable of controlling metastatic and proliferative potential of NSCLC by the way of the modification of IRE1α/XBP-1 signaling in tumor-associated macrophages.

## INTRODUCTION

Lung cancer has been described as the leading cause of cancer-related deaths globally and of all lung cancers, approximately 85% are NSCLC [1]. Both intracellular and membrane-bound PD-L1 expressing on NSCLC cells enhance cancer and chemotherapeutic resistance and promote immune evasion. Yet, little is understood pertaining to the molecular actions of the normal function of these proteins.

The role of tumor immune microenvironment has now been established to have a decisive role in the progression of NSCLC [2, 3], tumor-associated macrophages (TAMs) are characterised as the most prevailing immune cell population in the TME and reported to be related to poor prognosis in numerous tumour types [4]. The TME is characterized by local immune balance being maintained partly by M1 and mDC leaders that hence condone pro-inflammatory while suppressing immune responses. Recently, the unfolded protein response (UPR) is being considered as source of these events. Here, we show that IRE1α arm of the UPR is implicated in macrophage polarization *in vitro* as well as *in vivo* involving the up-regulation of IL-6, IL-23, arginase 1, CD86 and PD-L1. The IRE1α/X-box-binding protein 1 (Xbp1) pathway was also blocked pharmacologically or genetically deficient in the macrophages which exhibit reduced polarization and decreased CD86 and PD-L1 expression this regulation occurred in an independent manner of IFNγ signaling.

Based on this, cancer immunotherapies for the treatment of NSCLC are currently being developed in clinical practice [5, 6]. Among these drugs and therapies, the programmed death-1/programmed death ligand-1 (PD-1/PD-L1) blockade is a newly raised cancer immunotherapy for cancer treatment, and the related drugs are under development for the clinical treatment of NSCLC [7, 8]. These therapeutic agents include the PD-1 inhibitor navulizumab (Opdivo) [9, 10], pabolizumab (Keytruda) [11, 12], and the PD-L1 inhibitor atalizumab (Tecentriq) [13] and so on, and this study is mainly related to the investigation of this study with the help of navulizumab (Opdivo), and the effect of PD-L1 on IRE1α/XBP-1 signaling pathway.

## MATERIALS AND METHODS

### Experimental animal materials and reagents

A total of 32 8-12-week-old 129Sv/Ev homozygous female mice and 8-12-week-old C57BL/6 female mice were purchased from Henan Skibbes Bio-technology Co. Ltd (License No. SCXK (Yu)). All the animal studies were carried out in compliance with the protocols approved by the Institutional Animal Care and Use Committee (IA-CUC) of Fudan University (C2117250220 and C2020-0005). Lung cancer cells A549, Hcc827 cells and macrophages THP-1 were obtained from the Cell Bank of type C proast no of China, SBICAS (Shanghai Institute for Biological Science). Fetal bovine serum and RPMI 1640 medium were transported from Biyuntian Biotechnology Co., Ltd; nabulizumab and 4 μ8c inhibitor were purchased from Sidi Pharmaceutical Co. Ltd.; 4% paraformaldehyde fixative and crystal violet staining were purchased from Golden Clone Beijing Solepol Co. Ltd.; Western blot kits were purchased from Shanghai Xinyu Biotech Co. Ltd.; cell counter, 6-well culture plate, cell culture incubator and protein blotting system were purchased from Hangzhou Kerabo Biological Instrument Co.

### Mouse modeling

The primary tumor was established by subcutaneously injecting 0.5 × 10^6^ A549 cells in 100 μL of PBS into the right leg of mice. Subsequently, the secondary tumor was established by subcutaneously injecting 0.1 × 10^6^ A549 cells in 100 μL of PBS into the left leg. The *in vitro* response was observed. The first group served as the control group. In the second group, mice were treated with Nalwuliyoudan antibody twice a week via intraperitoneal injection (10 mg/kg) from the 5^th^ day after tumor cell injection until death. In the third group, mice were treated with the IRE1α inhibitor 4 μ8c, which was administered at a dose of 200 μg (100 μL) per mouse every 2 days for a total of 5 doses starting from the 5^th^ day after tumor cell injection until death. In the fourth group, mice were treated with both Nalwuliyoudan antibody (10 mg/kg) via intraperitoneal injection twice a week and the IRE1α inhibitor 4 μ8c (200 μg/100 μL) per mouse every 2 days for a total of 5 doses starting from the 5^th^ day after tumor cell injection until death. The mice were monitored daily for their status, mortality, and growth. The size of the subcutaneous transplant tumor in millimeters (a) and the width of the tumor in millimeters (b) were used to find the tumor volume. The harvested transplant tumor tissues were then immersed in 4% paraformaldehyde for a period of 48 hours, followed by dehydration and paraffin embedding for subsequent Hematoxylin and Eosin (HE) staining and immunohistochemical staining experiments. All animal experiments complied with the provisions of animal protection, animal welfare, animal ethics, the 3Rs rule and national experimental animal welfare and ethical regulation. The animal experimental procedures were fully compliant with approved guidelines and ARRIVE guidelines. The study was conducted and ethical approval was obtained from the Biomedical Research Ethics Committee in the Fourth Hospital of Hebei Medical University (SYXK (Ji) 2022-011). The steps for implanting A549 tumor cells into nude mice are as follows. Prepare A549 tumor cells: culture A549 cell line and let it reach the logarithmic growth phase. Collect cells, wash them with sterile PBS, and prepare cell suspension. Count the cells under a microscope and adjust the concentration of the cell suspension according to experimental needs. Prepare nude mice: select nude mice as experimental animals because they lack an immune system and can better accept xenografts. Ensure that the nude mice are healthy and meet the experimental requirements. Anesthetize the nude mice: use appropriate anesthesia to ensure that the nude mice do not experience any pain or discomfort during the surgical procedure. Prepare the surgical area: perform the surgery on the lateral abdomen or neck of the nude mice under sterile and disinfection conditions. You can use 75% alcohol or other disinfectants for disinfection. Cell implantation: use a syringe and fine needle to inject the A549 tumor cell suspension into the nude mice. You can choose to inject the cell suspension into subcutaneous tissue or deeper tissues such as muscle or fat layer. Suture the wound: close the surgical incision with sutures or adhesive tape to prevent infection and cell leakage. Postoperative care: after ensuring that the mice have recovered from anesthesia, keep them in a temperature-controlled environment to maintain stable body temperature. Observe the behavior and health status of the nude mice and provide appropriate food and water sources.

### HE staining

The above paraffin-embedded tissue sections were subjected to conventional xylene deparaffinization, sequentially dehydrated, washed with water, stained with hematoxylin for 5 min, rinsed again and then drained of water, differentiated using 1% hydrochloric acid ethanol solution. The samples were washed in PBS for 15 min, then rinsed with running water, stained with eosin solution for 2 min, and finally dehydrated until transparent, sealed with neutral resin and then used a microscope for picture acquisition and observation and analysis.

### Histochemical staining

The paraffin-embedded tissue sections were deparaffinized, then subjected to gradient alcohol dehydration, high-pressure repair of antigens, PBS washing of sections, inactivation of endogenous enzymes, PBS washing of sections, closure of the natural antibodies in the tissues, dropwise addition of primary antibody treatment. The goat serum on the sections was gently flung away, and the moisture of the surrounding tissues was sucked up with tissue paper, and dropwise addition of the appropriate amount of the primary antibody dilution of α-actin (1:500 dilution) was done; fully, evenly cover the tissue, and place it in a light-proof wet box at 4°C overnight. The next day, remove the wet box from the refrigerator and wash the sections with PBS. The washed sections were removed from the box, gently blotted with tissue paper, and the dilution of the fluorescent goat anti-rabbit secondary antibody (ab97080, 1:500) was added to sections and incubated for 45 min at room temperature and PBST was used as a wash. DAB color development: under the microscope, control the time of color development, the positive part of the dark brown, the remaining parts of the colorless water to terminate the reaction. Hematoxylin re-staining of cell nuclei, routine differentiation, return to blue, dehydration and transparency after neutral gum sealing were done. Observe under the microscope and take pictures.

### Cell culture

THP-1 cell line was purchased from the Cell Resource Center of Shanghai Institutes for Biological Sciences, Chinese Academy of Sciences. A549 cells and hcc827 cells were also purchased from the Cell Resource Center of Shanghai Institutes for Biological Sciences, Chinese Academy of Sciences and the cells were maintained in DMEM medium supplemented with 10% fetal bovine serum and cells were incubated at 37°C with 5% CO_2_. THP-1 cells were divided into four groups: the control group is meant to be a perfectly normal reference group with no treatments apart from routine observation, monoclonal antibody group with Nalwuliyoudan antibody pretreatment, inhibitor group with 4 μ8c pretreatment, and monoclonal antibody + inhibitor group with Nalwuliyoudan antibody and 4 μ8c pretreatment. Four groups of 24 h pretreated THP-1 cells were cultured with A549 cells. (Four groups of THP-1 cells were partly separated and cocultured with hcc827 cells). Specifically, 2 × 10^5^ logarithmic phase A549 cells (hcc827 cells) were seeded in 6-well plates, and after 24 hours, Transwell inserts with a pore size of 0.4 μm were placed in the wells. The inserts were then loaded with the four groups of THP-1 cells that had undergone different pretreatments for co-culture.

### Western blot

Afterwards, THP-1 cells or A549 cells were lysed by RIPA lysate to prepare total protein. The supernatant was then harvested by centrifugation at a speed of 10, 000 rpm and at 4°C. The protein content was measured using bicinchoninic acid assay and the samples were heated at 100°C for 10 min. After SDS-PAGE, the protein samples were equilibrated in transfer buffer for 15 min and then blotted onto PVDF membranes under constant current. This was followed by washing the membrane with 5% TBST skim milk powder for an hour at RT and the addition of the primary antibody at 4°C for a whole night. The following day, the secondary antibody was applied to ensure that the protein bands were reacted with the secondary antibody at the room temperature. These membranes were washed 3 times and then stained and visualized using an imaging system. Finally, the protein bands were densitometrically analyzed.

### Scratch experiment

A549 cells (hcc827 cells) from the above four groups were inoculated into 6-well plates and cultured at 37°C, 5% CO_2_. After the cells were spread all over the bottom of the plate, a uniform scratch was made perpendicular to the bottom of the plate behind the plate, and aspirated the cell culture medium after the test: cleansed the cell debris under the formed scratch using PBS. The cells were removed from the plate after 48 h of incubation, and the re–epithelialization of the scratches was studied and photographed under an inverted microscope, and the size of the healing area of the scratches was analyzed by ImageJ Software.

### Transwell experiments

Subsequently, the diluted Matrigel gel was coated, the surface of the upper chamber of the Transwell, and the matrix barrier layer were cultured at 37°C. The density of A549 cells (hcc827 cells) in each group was adjusted to 2 × 10^4^ /mL with fresh culture medium without fetal bovine serum, 200 μL of suspension was transferred to the upper chamber, and 600 μL of fresh culture medium containing fetal bovine serum was introduced to the lower chamber. After 24 h of incubation, the chambers were taken out and cells that did not penetrate the membrane were peeled off; the samples were then fixed in 4% paraformaldehyde and stained with 0.1% crystalline violet for 10 min, viewed and photographed using a light microscope and membrane penetration, i.e., the number of invasions, was counted in each group.

### CCK-8 method and clone formation experiments

CCK-8 method: A549 cells (hcc827 cells) were washed using sterile PBS to remove the cell layer from the culture dish. Add appropriate amount of sterile trypsin (Trypsin) solution to dissociate the cell layer. Add the same volume of medium and gently shake the dissociated cells to prepare a cell suspension. Transfer the cell suspension to a new Petri dish and add new medium. Put into the incubator to continue the culture. Then the A549 cells (hcc827 cells) obtained from the culture were inoculated in 96-well plates at a density of 2000 cells/100 μl/well, and put into the cell culture incubator at 37°C, 5% CO_2_ for further cultivation. When the cells were completely attached to the wall, the control group was changed to normal complete medium, and the experimental group was changed to complete medium containing the drug and continued to be cultured in the incubator. The absorbance (OD) values at 450 nm were measured after the cells were changed to serum-free medium containing 10% CCK8 reaction medium at 0 h, 24 h, 48 h and 72 h respectively, and the results of the four experiments were summarized in total.

Clone formation experiment: A549 cells (hcc827 cells) are plated at 700 cells/2 ml/well and incubated in a cell culture incubator. After the cells were completely attached to the wall, The control was switched to normal complete medium while the experimental was switched to complete medium plus the drug, and the medium was changed every 2d to continue the culture. After 6d–10d of incubation until cell colonies were formed, the culture medium in all wells was aspirated and discarded, 1 ml of 4% paraformaldehyde was added to each well after PBS washing for 30–60 min, 1 ml of crystal violet staining was added to each well after PBS washing for 10–20 min, and after washing with PBS for several times, the cells were counted and photographed after air-drying (each well was photographed individually), and the results of the four experiments were summarized in the results of the four experimental groups.

### Statistical data analysis

All data were statistically analyzed using GraphPad Prism 5.0 software. The experimental data of each group were expressed as mean ± standard deviation (SD). Comparisons between groups were performed using *t*-test. *p* < 0.05 was considered statistically significant difference.

## RESULTS

### PD-L1 monoclonal antibodies and IRE1α inhibitors are also effective in suppressing NSCLC tumors

**Figure 1:**
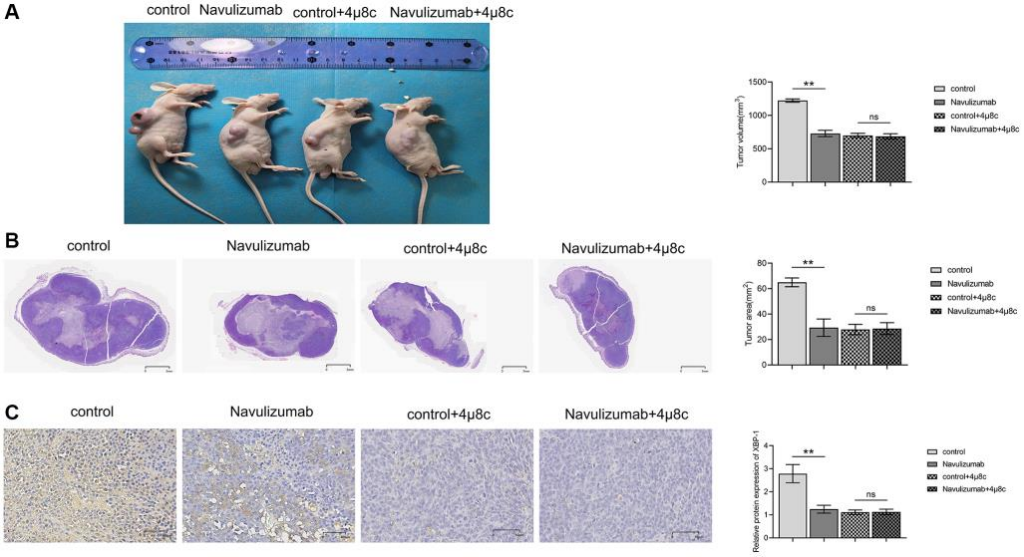
**PD-L1 monoclonal antibodies as well as IRE1α inhibitors have inhibitory effects on NSCLC tumors.** (**A**) Comparison and statistics of tumor volume size in each group. (**B**) Comparison and statistics of tumor area size in each group. (**C**) Comparison and statistics of indicator P-IRE1α and XBP-1 in each group. (^*^P < 0.05; ^**^P < 0.01).

The findings of the experiment revealed that the size of the tumor was considerably small in the animals that underwent nabulizumab intervention as compared to the control animals; however, no variation was seen in animal groups with IRE1α inhibitor treatment, as shown in Figure 1A below. HE staining results revealed that the tumor was largest in the control group, and smaller in the nabulizumab intervention group and in the two groups administered with IRE1α inhibitor in the NSCLC model of mice; in Figure 1A, the raw data of the gene expression are presented and in Figure 1B the final quantification of the results appears. Analysis of the data obtained from histochemical staining indicated that the indexes of P-IRE1α and XBP-1 were the highest in the control group and lesser in the nabulizumab intervention group, and both indexes were lower in the two groups that added 4 μ8c; the results are shown in Figure 1C.

### Indicators of respective related proteins in THP-1 and A549 cells

**Figure 2:**
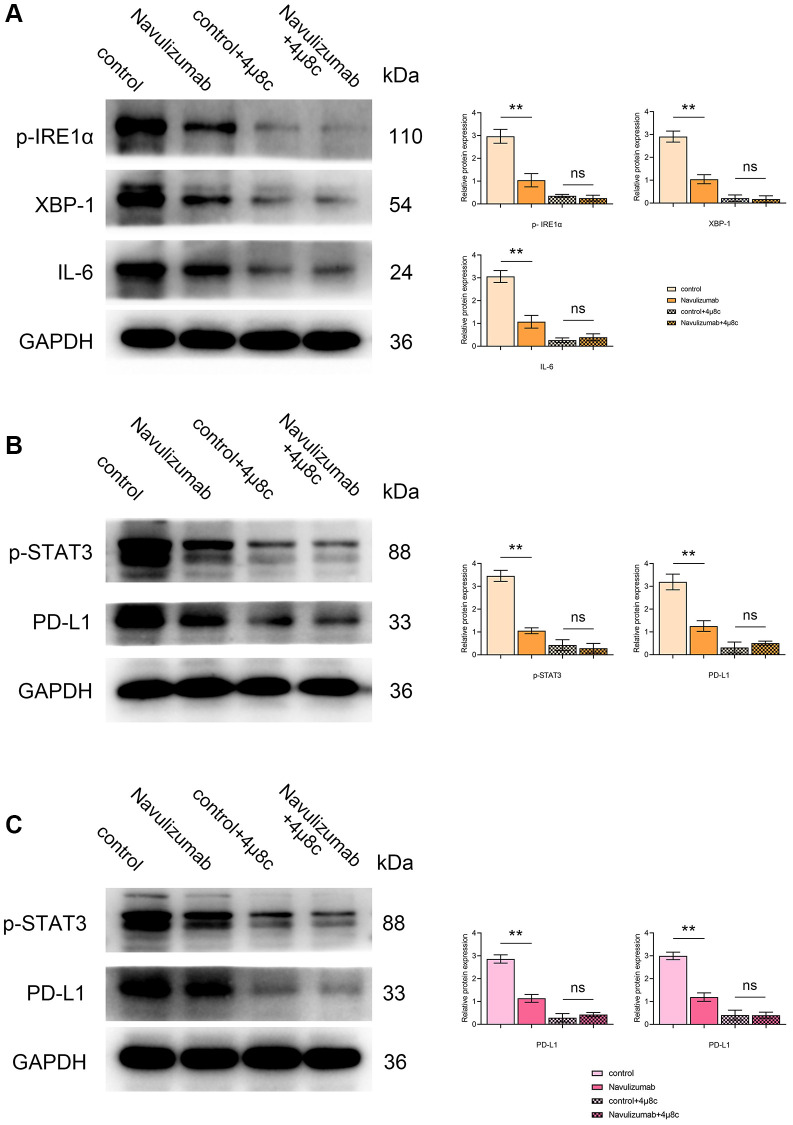
**Effect of nabulizumab and IRE1α inhibitor on protein metrics in different cells.** (**A**) Protein indexes and statistics of P- IRE1α, XBP-1s, IL-6 in THP-1 cells in each group. (**B**) Protein indexes and statistics of P-STAT3, PD-L1 in THP-1 cells in each group. (**C**) Protein indexes and statistics of P-STAT3, PD-L1 in A549 cells in each group. (^*^P < 0.05; ^**^P < 0.01).

Western blot results showed that P- IRE1α, XBP-1s, IL-6, P-STAT3 and PD-L1 in THP-1 cells in the nabulizumab intervention group were significantly lower than those in the control group, whereas those in the 4 μ8c treatment group were significantly lower than those in the A549 cells in the nabulizumab intervention group. The results of the nabulizumab intervention group are shown in Figure 2A, 2B below; there was a lower score of P-STAT3 and PD-L1 indexes in nabulizumab-treated A549 cells than in control cells, while the score was higher in 4 μ8c-treated cells than in control cells, and the results are shown in Figure 2C below.

### The situation of related protein indexes in THP-1 cells and the migration and invasion of A549 cells (hcc827 cells)

**Figure 3:**
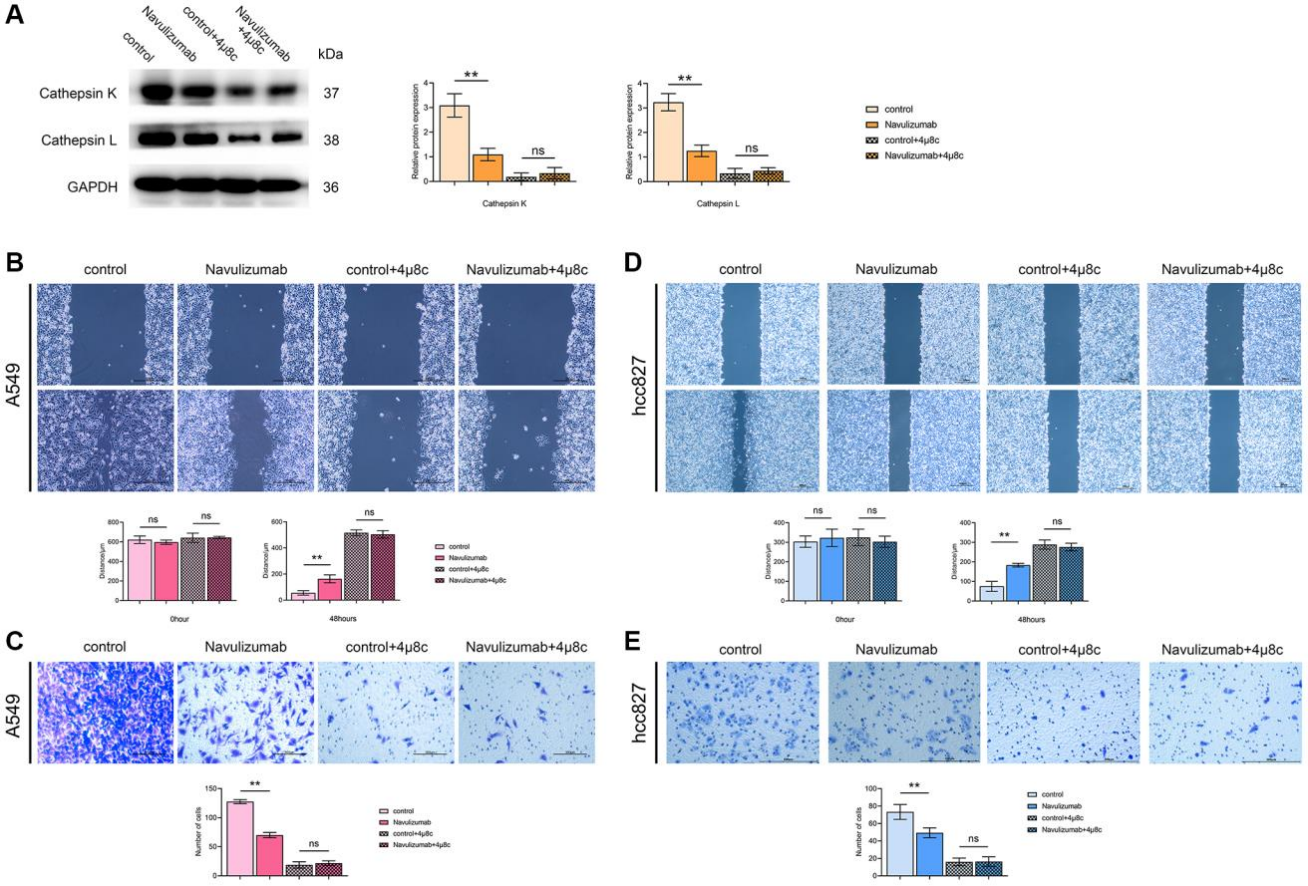
**The situation of related protein indexes in THP-1 cells and the migration and invasion of A549 cells (hcc827 cells).** (**A**) Protein indexes and statistics of Cathepsin K and Cathepsin L in THP-1 cells in each group. (**B**, **D**) Picture comparison and statistics of A549 cell (hcc827 cells) migration at 0 h and 48 h in the four groups. (**C**, **E**) Picture comparison and statistics of A549 cell (hcc827 cells) invasion in the four groups. (^*^P < 0.05; ^**^P < 0.01).

The levels of nabulizumab intervention groups of THP-1 cells were significantly reduced than those of the control group by the results of Western blot assay of Cathepsin K and Cathepsin L indexes, and higher in the 4 μ8c-treated group than in the nabulizumab-intervention group; the outcomes are depicted in the Figure 3A. The results of the scratch experiment showed that the scratch healing area was narrow in the control group, the results of the tests specific to width are presented in the figure below: nabulizumab intervention group: medium; IRE1α inhibition group: wide; nabulizumab intervention + IRE1α inhibition group: wide; Figure 3B, 3D). As seen in the Transwell assay, the number of cellular invasions in the control groups was significantly high; the number of cellular invasions in the nabulizumab intervention group was medium; the number of cellular invasions in the control group was high; the number of cellular invasions in the control group was high; and the number of cellular invasions in the control group was low. The incidence of cell invasion was considered moderate; less cell invasion was present in both IRE1α inhibition and nabulizumab intervention + IRE1α inhibition groups; the results are shown in Figure 3C, 3E.

### The situation of related protein indexes in THP-1 cells and proliferation of A549 cells (hcc827 cells)

**Figure 4:**
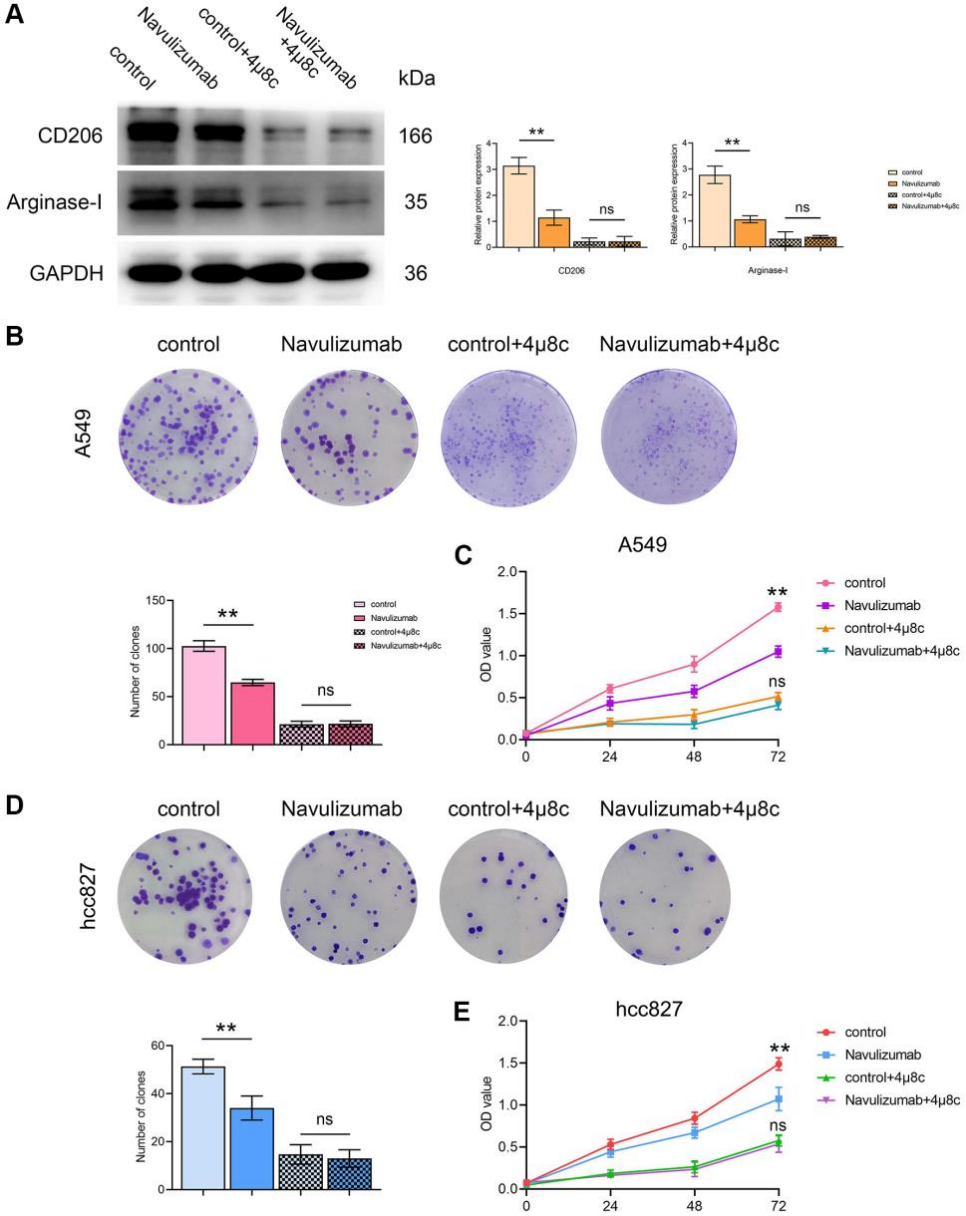
**The situation of related protein indexes in THP-1 cells and proliferation of A549 cells (hcc827 cells).** (**A**) Protein indexes and statistics of CD206 and Arginase-I in THP-1 cells in each group. (**B**, **D**) Proliferation picture and statistical comparison of four groups of A549 cells (hcc827 cells) in clone formation assay. (**C**, **E**) Proliferation picture and statistical comparison of four groups of A549 cells (hcc827 cells) on the CCK-8 assay. (^*^P < 0.05; ^**^P < 0.01).

The determination of Western blot assay pointed out that the CD206 and Arginase-I indexes were up-regulated in THP-1 cells of the nabulizumab-intervention group compared with the control group, and higher in the 4 μ8c-treated group than in the control group; the results are expressed in Figure 4A. Clone formation assay was performed to determine cell proliferation and it was observed that the percentage of cell proliferation was high in the control group., medium in the nabulizumab intervention group, and low in the IRE1α inhibition group and the nabulizumab + IRE1α inhibition group; the results are expressed in Figure 4B, 4D. The results of CCK-8 assay showed that the cell proliferation was high in the control group, medium in the nabulizumab intervention group, and medium in the IRE1α inhibition and nabulizumab intervention groups. IRE1α inhibition group and nabulizumab intervention + IRE1α inhibition group both had a low number of cell invasion; the results are shown in Figure 4C, 4E.

## DISCUSSION

NSCLC is a kind of lung cancer, and its main clinical manifestations are cough, chest pain, dyspnea, etc. Surgical resection is generally selected for early NSCLC with good efficacy, but most patients are diagnosed as advanced stage, and chemotherapy is commonly used for advanced NSCLC. DOC is a common chemotherapy drug, which can inhibit the interphase cell function and affect cell mitosis by interfering with the microtubule network, and reduce tumor neoplasia. However, due to the large toxic side effects and the small inhibition of tumor immune escape, the tumor killing effect is small, making the treatment effect less than ideal.

Immunotherapy also has the advantages of strong targeting and small side effect for it has just emerged as a new treatment method instead of chemotherapy or traditional therapy. Immunotherapy prevents the tumor growth of malignancies by altering the profiles of TME [14]. One study demonstrated that ‘TAMs are responsible for the sustainment of PD-L1 expression in TME even if PD-L1 is lost on tumor cells’ [15]. Another study reveals that PD-L1 is present on TAMs of NSCLC patients [16]. Out of all the NSCLC patients, there are slightly more NSCLC patients with PD-L1 positive on TAMs than NSCLC patients with PD-L1 negative on TAMs. Thus, the analysis of PD-L1 on TAMs reveals them as a new target for cancer immunotherapy [17]. On such basis, it was established that TME controls the sensitivity of lung cancer to improve PD-1/PD-L1 blockade therapy attributed to the fact that the response of cancer cells to immunotherapy depends on TME.

A detailed study of the role of tumor-associated macrophages (TAMs) in the immunosuppressive function within tumors proposed that ER stress is critical for retention of the TAM population and modulating the ER. It is well understood that unfolded protein response (UPR) can control three unique proteins which are, y inositol-requiring enzyme 1 (IRE1), activating transcription factor 6 (ATF6), and protein kinase RNA-like to keep its inactive or bound to their membrane with GRP78/BiP. In this study, we mainly focus on inositol-requiring enzyme 1 (IRE1). IRE1 alpha is a homodimeric type I membrane protein which is resident in the lumen of the endoplasmic reticulum. After the activation of UPR, IRE1 alpha, self-dimerization and self-phosphorylation guarantee its activation (phospho-Ser7). After the above process, unfolded proteins accumulate to produce endoplasmic reticulum stress (ERS), and XBP-1 can directly bind to the promoter of the macrophage IL-6 gene to exert its effects, leading to overexpression of IL-6. IL-6 can act on JNK2 to phosphorylate P-STAT3, it also leads to production of CD2O6, Arginase-I, and PD-L1 that supplies nutrition to tumors contributing to its growth. CD2O6 and Arginase-I are reported to operate in non-small cell lung cancer cell and that they enhance their proliferation. TAMs secrete PD-L1 and the IL-6 generated by TAMs fosters P-STAT3 in non-small cell lung cancer and therefore honed PD-L1 in non-small cell lung cancer. Therefore, the PD-L1 produced by both the TAMs and the non-small cell lung cancer impact the TAMs cells. PD-L1 can also phosphorylate IRE1α, thereby promoting the action of the cysteine protease Cathepsins, which invade non-small cell lung cancer.

Nivolumab is a new immunotherapy drug and human immunoglobulin G4 monoclonal antibody. It has the ability to bind PD-1 receptor, prevent the binding of PD-L1 and PD-L2 and also antagonise the immunosuppressive function of PD-1 pathway. It can effectively block the binding of cancer cells and T cells, restore the normal physiological function of T cells, improve the recognition ability of T cells to cancer cells, play the clearance effect of T cells on cancer cells within the tumor microenvironment, and then reconstruct the anti-tumor immune response of the body, reduce the immunosuppressive effect of chemotherapy drugs, reduce the damage of chemotherapy on T lymphocyte subsets, and enhance the immune function of the patient by improving the activity of natural killer cells, body tolerance and NK cell activity. In addition, it is able to reinforce the effect of the immune system to eliminate tumor through blocking the effect of tumor cells on the inhibition of immune system, anti-angiogenesis, so as to achieve the intention of tumor treatment. Nivolumab has therapeutic impacts on numerous kinds of cancers, therefore it has influence on NSCLC. The inhibitory effect of IRE1α inhibitor 4 μ8c on IRE1α has a certain inhibitory effect on the progress of non-small cell lung cancer, and can be combined with nivolumab to strengthen the effect.

**Figure 5:**
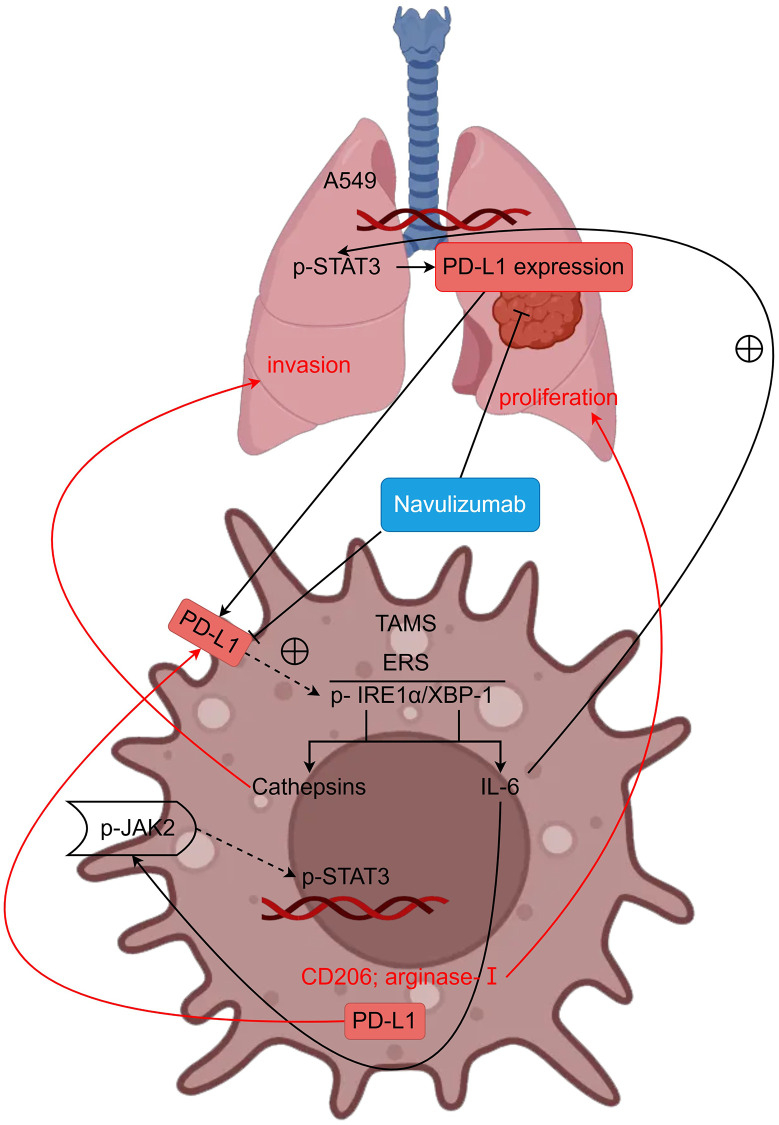
PD-L1 can regulate metastasis and proliferation of non-small cell lung cancer by modulating the IRE1α/XBP-1 signaling pathway in tumor-associated macrophages (schematic mechanism).

In summary, nivolumab can inhibit the metastasis and proliferation of NSCLC by inhibiting PD-L1 and regulating the IRE1α/XBP-1 signaling pathway in tumor-associated macrophages (Figure 5). This provides an experimental basis for clarifying the mechanism of inhibiting metastasis and immune escape of NSCLC. Of course, the mechanism of TAMs and PD-L1 expression in NSCLC cells needs further exploration and study.
